# Sanfilippo type A: new clinical manifestations and neuro-imaging findings in patients from the same family in Israel: a case report

**DOI:** 10.1186/1752-1947-8-78

**Published:** 2014-02-28

**Authors:** Rajech Sharkia, Muhammad Mahajnah, Abdelnaser Zalan, Chrysovalantis Sourlis, Peter Bauer, Ludger Schöls

**Affiliations:** 1 The Triangle Regional Research and Development Center, P. O. Box-2167, Kfar Qari' 30075, Israel; 2 Beit-Berl Academic College, Beit-Berl 44905, Israel; 3 Child Neurology and Development Center, Hillel-Yaffe Medical Center, 38100 Hadera, Israel; 4 Rappaport Faculty of Medicine, Technion, 31096 Haifa, Israel; 5 German Research Center for Neurodegenerative Diseases (DZNE), 72076 Tübingen, Germany; 6 Institute of Medical Genetics and Applied Genomics, University of Tübingen, 72076 Tübingen, Germany; 7 Department of Neurodegenerative Diseases and Hertie-Institute for Clinical Brain Research, University of Tübingen, Hoppe-Seyler Str. 3, 72076 Tübingen, Germany

**Keywords:** Mucopolysaccharidosis type III, Sanfilippo type A, T139M mutation, Israeli Arabs

## Abstract

**Introduction:**

Sanfilippo syndrome type A (mucopolysaccharidosis IIIA - MPS IIIA) is an autosomal recessive lysosomal storage disorder caused by a deficiency in sulfamidase.

**Case presentation:**

Two daughters (13 and 11 years old) of a consanguineous Palestinian family from the Israeli Arab community were investigated clinically and genetically for the presence of progressive neurodegenerative disease, psychomotor retardation and behavioral abnormalities. Development was normal up to one year of age. Thereafter, progressive motor and speech delay started. Metabolic screening including glycosaminoglycans, karyotype testing and magnetic resonance imaging were normal. Later in the disease, they developed severe spasticity and intellectual disability with autistic features and incontinence. Magnetic resonance imaging revealed diffuse hypomyelination with thinning of the corpus callosum. Genetic examination through whole exome sequencing revealed a homozygous mutation c.416C >T (p.T139M) in the *N-sulfoglucosamine sulfohydrolase* (*SGSH) gene*. Repeated biochemical testing at age 11 and 13 revealed increased levels of glycosaminoglycans confirming the diagnosis of Sanfilippo syndrome type A.

**Conclusion:**

These cases were considered to be the first report of Sanfilippo syndrome in Israel. We recommend that if similar clinical features are present during childhood, it is preferred to go directly and primarily for a genetic diagnosis of Sanfilippo syndrome, then secondarily for other lysosomal storage disorders that may also be involved.

## Introduction

The mucopolysaccharidoses (MPSs) are a group of seven inherited metabolic disorders characterized by the deficiency of one of the lysosome enzymes catalyzing the degradation of glucosaminoglycans (GAG) or mucopolysaccharides [1,2]. This deficiency leads to abnormal accumulation of GAG in the lysosomes which in turn gets excreted in the urine. GAG accumulation results in physical and mental handicap [3]. Mucopolysaccharidosis type III (MPS III) is known as Sanfilippo syndrome. It shows autosomal recessive inheritance and is caused by a deficiency of one of four enzymes involved in the degradation of the glucosaminoglycan heparan sulfate [4]. According to the defective enzyme, four subtypes of MPS III are designated as type A, B, C and D. The frequency of these subtypes varies between 0.28 and 4.1 per 100,000 live births [5]. It was found that Sanfilippo syndrome type A (MIM 252900) was the most frequent subtype accounting for 1 in 114,000 live births [6]. It is caused by a deficiency of the enzyme sulfamidase (heparan N-sulfatase), that degrades heparan sulfate; this causes an increase in the urinary excretion of heparan sulfate. Two biochemical tests were employed for MPS III diagnosis: (i) measuring the concentration of GAGs in urine, and (ii) measuring the enzyme activity in leucocytes or cultured fibroblasts [7,8]. The gene encoding sulfamidase (*N-sulfoglucosamine sulfohydrolase - SGSH*) is localized to chromosome 17, spans 11kb, contains 8 exons and encodes a protein of 502 amino acids [9]. There are 115 disease-causing *SGSH* mutations reported so far [1].

Clinical features and disease severity in patients with Sanfilippo syndrome type A vary considerably. A comprehensive study describing the clinical features of 92 patients suffering from MPS IIIA found a wide phenotypic variability and revealed a significant genotype and/or phenotype correlation [4].

As a part of a study on genetic diseases in consanguineous families of the Arab community in Israel, we investigated two patients with developmental delay starting between the first and the second year of life and progressing to severe psychomotor handicap at 10 years of age. Since extensive work-up, including glycosaminoglycans, was negative, diagnosis of Sanfilippo syndrome type A was only made by next-generation whole exome sequencing. To the best of our knowledge, the two patients in our study were the first Israeli cases to be reported in the literature.

## Case presentation

This research was prospectively reviewed and approved by the ethics committee in our center. Two siblings of a consanguineous Palestinian family from the Israeli Arab community attended the child development and pediatric neurology clinic due to progressive psychomotor retardation and behavioral difficulties.

### Patient 1

Our first patient is a 13-year-old girl who was born to consanguineous parents (first degree cousins) after a full term pregnancy by cesarean section delivery due to maternal hypertension, pre-eclampsia and breech presentation with a normal birth weight of 3.3Kg. According to her parents, she had normal development till the age of one year. She was first referred to a pediatric neurology clinic at the age of 18 months for suspected motor delay. At this time, her examination revealed no dysmorphic features and no visceral enlargement. She showed mild motor delay but could stand with support, and could speak up to five words. Her neurologic examination revealed hypertonia of the lower extremities and increased tendon reflexes of the lower and upper limbs. The Babinski sign was flexor. Cranial nerves, sensory perception and cerebellar function were normal. She was referred for metabolic screening, including glycosaminoglycans in the urine (u-GAGs), which was normal. Furthermore, karyotype testing and brain magnetic resonance imaging (MRI) were carried out at the age of two years and found to be normal. She also underwent cerebrospinal fluid examination for protein, glucose, lactate levels and cell count yielding normal results.

**Figure 1:**
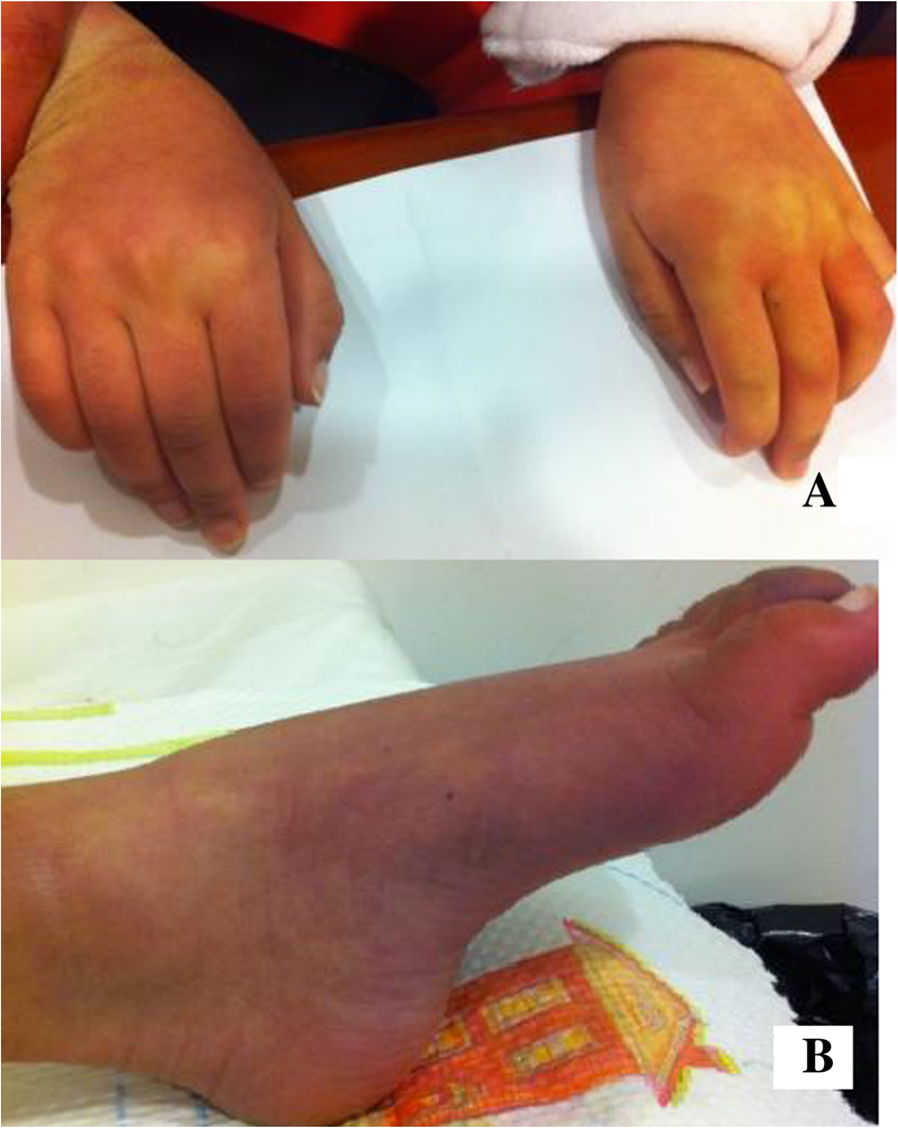
(A): Patient 1, clear skin discolorization, (B): Patient 2, mild pes cavus and skin discolorization.

**Figure 2:**
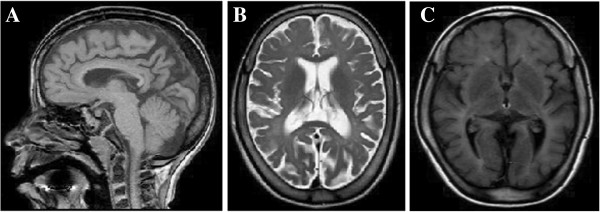
(A, B, C): Magnetic resonance imaging of Patient 1, diffuse hypomyelienation, thinning of the carpus callosum and moderate cerebral atrophy.

She was further followed-up by the pediatric neurology and child development clinic. At the age of three years, a second u-GAGs screening was performed and found to be normal. At the age of 11 years, she started to suffer from recurrent convulsions; paroxysmal events of generalized hypertonia, and absences with loss of consciousness for several minutes. The electroencephalography (EEG) record was normal but she was treated with valporic acid with good response. At the age of 12 years, her neurologic signs were progressive, including severe spasticity, impaired dexterity and severe intellectual disability with autistic features and incontinence. She also had hearing impairment (auditory evoked potentials were abnormal on both sides), joint contractures, pes cavus, and skin discoloration of the arms, hands, legs and feet (Figure 1). A second brain MRI revealed parieto-occipital atrophy, including cortex atrophy, thin corpus callosum, white matter thinning and mild ventriculomegaly (Figure 2).

### Patient 2

The second patient is an 11-year-old girl, who is the younger sister of Patient 1. She was born after a full term pregnancy by cesarean section delivery due to maternal hypertension, pre-eclampsia and breech presentation with normal birth weight (3.0Kg). According to her parents, her development was normal until the age of 18 months. Then she was referred to pediatric neurology for suspected motor and speech delay. At this age, her examination revealed neither dysmorphic features nor visceral enlargement. She showed mild motor and speech delay. Her neurologic examination revealed similar findings to her elder sister with hypertonia of the lower extremities and increased tendon reflexes of upper and lower limbs. Babinski sign was negative. Cranial nerves, sensory reactions and cerebellar function were normal. Metabolic screening including u-GAGs was normal. Furthermore, karyotype testing and cerebrospinal fluid examination for protein, glucose, lactate levels and cell count yielded normal results.

She continued to deteriorate with progression of neurologic symptoms and developmental deficits. At age 11 years she presented with similar findings as her elder sister, including severe spasticity, impaired dexterity and severe intellectual disability with autistic features, joint contractures, pes cavus, and skin discoloration of the arms, hands, legs and feet (Figure 1). Brain MRI revealed similar findings as in Patient 1, including diffuse hypomyelination with thinning of the corpus callosum.

In light of these findings, genetic tests were sought by the parents for family planning purposes. Homozygosity mapping was undertaken by single nucleotide polymorphism (SNP) microarray genotyping (Illumina 6k human mapping array, Illumina, San Diego, CA, USA) in both affected siblings and the parents. Eight regions of shared homozygosity in the two sibs spanning 84MB in total were revealed. Exome sequencing (NimbleGen SeqCap V2, Roche, Mannheim, Germany) followed by Illumina GAIIx paired-end sequencing for 2×76bp (Illumina, San Diego, CA, USA) uncovered a homozygous mutation c.416C > T, p.T139M in the *SGSH* gene causing mucopolysaccharidosis type IIIA, that is, Sanfilippo A syndrome. This mutation has already been discussed in a published article [10]. A third u-GAG test was performed, which revealed an increased level of u-GAGs, thus, confirming the genetic results.

## Discussion

There are four subtypes of mucopolysaccharidosis III (MPS III = Sanfilippo syndrome), namely A, B, C and D, that are caused by deficiency of one of four lysosomal enzymes, respectively. All are inherited by an autosomal recessive trait; signs, symptoms and the course of the disease in the different subtypes are indistinguishable. Therefore, misdiagnosis of the various subtypes often occurs [11]. MPS IIIA is caused by a deficiency of the enzyme sulfamidase due to *SGSH* mutations, which is involved in the stepwise degradation of heparan sulfate. *SGSH* is characterized by the presence of multiple polymorphisms [9].

The summary of clinical signs and symptoms in our reported cases, compared with other patients described in previous studies [1,4,5] are presented in Table 1. Most of the MPS IIIA symptoms found in our patients were similar to the described patients. These include normal development until one year of age followed by delay of motor and speech development as well as behavioral problems and facial dysmorphisms. According to the clinical progression, severe, intermediate and attenuated courses are differentiated. The clinical features of our two patients indicated a severe course, based on their full dependence on external care, complete loss of initiative and restriction to wheelchairs. However, other patients with Sanfilippo type A had even more rapid progression of symptoms [5].

**Table 1:** **Comparing symptoms of Sanfilippo type A as described in the literature**[1,3,4]**with our patients**

**Clinical features**	**Reported phenotype**	**Patient-1**	**Patient-2**
Pregnancy and delivery complications:	35 to 43 weeks, birth weight: 3534g, maternal hypertension, mild bleeding, normal delivery for majority of patients.	Normal pregnancy, birth weight: 3300g, delivery by caesarean section, maternal hypertension, pre-eclampsia and breech presentation.	Normal pregnancy, birth weight: 3000g, delivery by caesarean section, maternal hypertension, pre-eclampsia and breech presentation.
First signs and symptoms:	Normal development until one year, later on developmental and speech delay with behavioral problems.	Normal development until one year, later on motor and speech delay.	Normal development until 1.5 years, later on motor and speech delay.
	Facial dysmorphisms.	Negative.	Negative.
	Hepatomegaly.	Negative.	Negative.
	Recurrent diarrhea.	Negative.	Negative.
	Recurrent ear, nose and/or throat (ENT) infections.	Negative.	Negative.
Age of diagnosis:	Median age of 4 years (range 2 to 47 years).	12 years.	10 years.
Behavioral problems:	Before 10 years of age: restlessness, temper tantrum, crying fits, hyperactivity, destructive and compulsive behavior and complete loss of initiative in the majority of patients.	At age three to four years: restlessness.	At age three to four years: restlessness.
		At age 10 to 11 years: complete loss of initiative with autistic-like behavior.	At age 10 to 11 years: complete loss of initiative with autistic-like behavior.
Sleeping problems:	Onset at a median age of 4 years (range 0 to 35 years) with difficulties of falling asleep and frequent nocturnal wakening.	No sleep disturbance.	No sleep disturbance.
Hearing problems:	Present in about 40% of patients.	Moderate to severe sensory bilateral hearing loss; abnormal auditory potentials (BAER).	Moderate to severe sensory bilateral hearing loss; abnormal auditory potentials (BAER).
Visual problems:	Formal visual testing not possible.	Formal visual testing not possible.	Formal visual testing not possible.
Fundoscopy:	Retinitis pigmentosa one-third of patients older than 21 years.	Normal.	Normal.
Epilepsy:	About 66% developed epilepsy at a median age of 11 years (range 1 to 43 years), but well controlled by medication.	Onset of epileptic seizure at the age of 11 years but well controlled by valproic acid.	None.
Age at death:	Median 18 years (range 6 to 59).	Current age 13 years.	Current age 11 years.
Joint contractures:	Present.	Present.	Present.
Scoliosis:	Present.	Present.	Present.
Kyphosis:	Present.	Present.	Present.
Pes cavus:	Not described.	Present.	Present.
Skin discoloration:	Not described.	Present.	Present.
Dysmorphic features:	Present.	Obvious at 10 to 11 years of age.	Obvious at 10 to 11 years of age.
Magnetic resonance imaging (MRI) findings:	Not reported.	Diffuse hypomyelination, thin corpus callosum and progressive cerebral atrophy.	Diffuse hypomyelination and thin corpus callosum.

Between the age of 10 and 11 years: autistic-like behaviors (according to Diagnostic and Statistical Manual of Mental Disorders (DSM) IV criteria), were found in both our patients. This symptom was not observed in a large series of MPS IIIA [4] but was described in our patients at the age of three to four years in another study [11]. On the other hand, some previously described clinical symptoms like hepatomegaly, recurrent diarrhea and recurrent ear, nose and/or throat (ENT) infections [4], were not observed in our patients. Furthermore, we observed pes cavus and skin discoloration in our patients that were not reported before in patients with Sanfilippo syndrome type A. Both signs may be caused by neurodegeneration due to the production of neurotoxic substances [12]. It was previously noted that evolving joint pain and joint contractures in the absence of inflammation should always raise the suspicion of an MPS disorder [13]. Additionally, both patients were born after complicated pregnancy with hypertension and pre-eclampsia. Previously, it was reported that only 6 cases from 76 Sanfilippo A patients were born after complicated pregnancy, such as hypertension and mild bleeding, but pre-eclampsia was not reported [4].

Mucopolysaccharidosis III disease is characterized by an inability to degrade heparan sulfate that leads to its excretion in the urine. The assay of urine-GAG is usually the first step in biochemical diagnosis of this disease. U-GAG testing is easy to perform but it remains unreliable in the diagnosis of MPS III. Among the MPSs, patients with MPS III have comparatively lower levels of u-GAG, leading to some risk of false-negative results [14]. With respect to this problem, u-GAGs were determined twice in our patients but both times with normal results. Only the third test, after genetic diagnosis, revealed that u-GAG levels were higher than normal. Recently, it was found that urinary levels of heparan sulfate among patients with attenuated MPS IIIA are substantially lower than that in patients with severe MPS IIIA [15]. However, our patients had normal urinary heparan sulfate long into the disease despite a severe course of MPS IIIA. Thus, normal u-GAG levels (that is, negative u-GAG testing) do not rule out the diagnosis of MPS III.

MRI revealed progressive parieto-occipital atrophy, including cortex atrophy, thin corpus callosum white matter thinning due to diffuse hypomyelination in our patients. These findings were not previously described in patients with type A of Sanfilippo syndrome but only in other Sanfilippo subtypes [3]. Neuronal degeneration is probably a late phenomenon, as cortical atrophy was not observed in the early MRI scans in our patients [16].

Recently, a therapeutic approach to Sanfilippo disease has been proposed. Genistein is a naturally occurring isoflavone and inhibits heparan sulfate synthesis in cultured fibroblasts from MPS III patients. Genistein treatment reduced plasma levels of heparan sulfate as well as urinary GAG excretion but failed to ameliorate clinical disease in Sanfilippo patients in a placebo-controlled crossover study [17]. Higher dosage may be more efficient but controlled trials have to be awaited. They should also help to clarify whether response to Genistein is dependent on the stage of the disease.

The mutation found in our patients (p.T139M) was described previously as a cause of MPS IIIA in a patient from the UK but no clinical description was reported [10]. Here we show that the T139M mutation in *SGSH* can cause a severe phenotype and does occur in the Arab population of Israel.

As the parents insisted on having a healthy child, and due to their *status quo* as a consanguineous couple, it was inevitable to refer them to a genetic counseling unit and the PGD (pre-gestational diagnosis) clinic. Previous studies confirmed the correlation between the high prevalence of autosomal recessive genetic disorders and the high rate of consanguineous marriages in the Arab population [18].

## Conclusion

This study is considered to be the first report that described two patients with Sanfilippo syndrome in Israel. While the biochemical testing proved to be misleading for the proper diagnosis of Sanfilippo syndrome, it is recommended that if similar clinical features are present during childhood, it is preferred for the patients to go directly and specifically for genetic diagnosis of Sanfilippo syndrome, though, other lysosomal storage disorders may also be involved. Furthermore, MRI imaging revealed diffuse hypomyelination with thinning of the corpus callosum that might be associated with this syndrome.

## Consent

Written informed consent was obtained from the parents of our patients for publication of this case report and the accompanying images. A copy of the written consent is available for review by the Editor-in-Chief of this journal.

## Abbreviations

GAG: Glucosaminoglycans; MPS III: Mucopolysaccharidosis type III; MPSs: Mucopolysaccharidoses; MRI: Magnetic resonance imaging; PGD: Pre-gestational diagnosis; U-GAG: Glycosaminoglycans in urine.

## Competing interests

The authors declare that they have no competing interests.

## Authors’ contributions

RS and LS conceived of the study, and participated in its coordination and contributed in writing the manuscript. MM carried out the clinical diagnosis and analyzed the patient data and helped in writing the manuscript. CS and PB carried out the molecular genetics approach and helped to draft the manuscript. AZ analyzed and interpreted the patient data and contributed in critical writing of the manuscript. All authors read and approved the final manuscript.
